# Arachnoid Inflammation

**Published:** 1822-03-01

**Authors:** 


### Medico-Chirurgical Review,

AND

### JOURNAL OF MEDICAL SCIENCE.

(Analytical Series.)

"Nec Aranearum textus ideò melior, quia ex se fila tingunt; nec noster vilior, quia ex alienis lubamus, ut apes."

VOL. II.] MARCH 1, 1822. [No. 8.

### I.

### ARACHNOID INFLAMMATION, CEREBRAL AND SPINAL.

*Recherches sur l'Inflammation de l'Arachnoide, Cerebrale, et Spinale; ou Histoire Theorique et Pratique de l'Arachnitis.* Ouvrage fait conjointement par PARENT-DUCHATELET, M. D. Medecin en Chef de la 4e Legion de la Garde Nationale, &c. et L. MARTINET, M. D. &c. &c. One large volume, 8vo, pp. 612. Paris, 1821.

——————— quid nobis certius ipsis

Sensibus esse potest, quo vera ac falsa notemus.—LUCRET.

THAT there cannot be a more important subject of investigation than that which occupies the volume before us, must be admittted by every one. The brain, as the organ which unites our moral and physical natures, exercises unbounded sway over our mental and corporeal faculties and functions; while its slightest deviations from health involve derangements of numerous other organs and structures in the living machine. These peculiarities of the sensorium, its appendages, and its coverings, render an investigation of their disorders so extremely difficult and perplexing, that nothing but the most ardent, unwearied, and systematic course of clinical observation and pathological research can be at all expected to throw light on the obscurity of the subject. Such clinical records and necrologic examinations have not been systematically pursued in this country; for the writings of Dr. Abercrombie and a few others are confessedly sketches, or contributions towards a more regular work on the important class of diseases now under consideration. It is a rare thing, in this country, to see two men uniting to watch and record the phenomena of disease at the bed side, and minutely examine, after death, the corresponding traces of disorganization. Such a process, especially when carried on in a great public hospital, under the eye of the medical officers and visiters, acquires an authenticity of inestimable value, and which places it beyond the humiliating sarcasm of Cullen, who observed—"that there are more false *facts* than false *theories* in medicine."

If Routinism and Apathy should raise their voices—and doubtless they will—against the arduous, or, in their apprehension, fruitless attempt to discriminate the diseases of structures so contiguous as the membranes and substance of the brain, we must not, on that account, be turned from the path of investigation which Reason, as well as experience of the past, points out as the only one likely to lead us from darkness to light—from doubt to certainty, or at least probability—from empyricism to science. Let it be remembered also, that as it is by minutely studying every *part*, we can hope to become intimately acquainted with the *whole*; so a rigid and careful examination of the disorders affecting each particular tissue enveloping the cerebral mass, must, of necessity, improve our general knowledge of encephalic disease, even when we are unable to determine the precise seat of lesion in the living body. On this account we must entreat the patient attention of our readers to the following analysis, which we trust and hope will amply repay the perusal. The extent of the article must be imputed to the size of the volume, the importance of the subject, and the improbability of any translation of the original into our language taking place.

It may be necessary to state, in limine, that almost all the observations contained in this work were made in the Hotel Dieu, under the eye of Dr. Recamier, and at the Hospice des Enfans, under the inspection of Drs. Jadelot and Nysten. The cases were carefully noted at the bed-side, and the histories can be verified by the hospital registers. The work itself has been submitted at the Institute, to the Royal Academy of Sciences, and a very detailed and very favourable report thereon has been drawn up by Portal, Dumeril, Pelletan, Hallè, and Baron Cuvier, concluding with the following sentence:—

> "Le travail dont nous venons de donner l'analyse, nous parait pouvoir contribuer à perfectionner la connaissance et le diagnostic, souvent bien difficiles, d'une maladie très-importante à bien caractériser, et par conséquent à assurer le succès de son traitement."

The approbation of such a distinguished body as the In-

STITUTE, is a certain passport to public attention; and, without farther preface, we shall proceed to an analysis of the work.

Our authors observe that the diseases to which the cerebral envelopes (dura mater, arachnoid, and pia mater) are subject, may be conveniently classed under the following heads: —1. Congestions. 2. Exhalations, serous or sanguineous. 3. Hæmorrhages. 4. Inflammations. 5. Organic affections, or changes of structure. Where the cerebral pulp itself is affected, "exhalations" must be omitted, and two other heads added—"commotion," and "nervous affections." The present enquiry of our authors is limited to the 4th head, viz. inflammation, and that of the middle envelope, or tunica arachnoides. Their reasons for this preference are, the obscurity in which arachnitis has hitherto been involved—the frequency and danger of the disease—the difficulty of the diagnosis—and, above all, the light which it throws on other cerebral affections.

1. The first chapter presents a neat, but very succinct sketch of the anatomy and physiology of the arachnoid,* cerebral and spinal. Our readers are aware that it is a delicate transparent membrane, closely in contact with the pia mater, and reflected, according to Bichat and other French anatomists, over the internal surface of the dura mater. Since Bichat's time, it is considered as passing in, to form a lining for all the ventricles of the cerebrum and cerebellum. It is constantly lubricated by a fine rosy exhalation, and is decidedly a *serous* membrane, performing the same functions in the head as the membranes investing the heart, lungs, and abdominal viscera, perform in their respective situations. In a healthy state it is insensible to touch or torture; but, when inflamed, it acquires a high degree of morbid sensibility —a change to which we must attribute the head-ache, more or less violent, but invariably attendant on arachnitis, forming its special character, and, in this respect, meriting the most profound attention of the practitioner. In its pathological as well as physical character, it harmonizes with the other serous membranes of the body. It is inflamed by the same causes which inflame them—and, like them, frequently throws out aqueous collections, or forms adhesions where the surfaces were before free. Finally, like the pleura and peritoneum, its mode of suppuration is by an effusion of whitish or sero-albuminous fluid, sometimes forming layers of false membrane, &c.

---

* For the sake of brevity, we shall use "*Arachnoid*" as a substantive throughout this article. It is so used by our authors.

II. The second chapter is on the important subjects of etiology and history of cerebral arachnitis.* Among the primary, most common, and most frequent causes of the disease, are percussions of the head,† insolation, organic lesions of the brain itself, apoplectic disposition, depressing passions of a moral nature. Among the secondary causes, in point of frequency or importance, are metastases of different kinds, suppression of habitual discharges, the use of strong drinks, and the common causes of other internal phlegmasiæ, as of pleuritis, gastritis, &c.

Our authors attach great importance to those percussions or commotions, which, though far too slight to produce fracture of the cranium, are yet capable of injuring the tender structure of the arachnoid.‡ It is curious that this cause determines the suppurative process in the arachnoid more frequently than any other cause. With the exception of two cases, the suppurative process was established in every instance where arachnitis resulted from external violence. To *insolation*, our authors were able to distinctly trace only two cases, probably because their patients were inhabitants of a great city, and little exposed to the direct rays of the sun. Doubtless in the country—and especially in warm countries—this cause is a very frequent one.

Our authors exhibit tabular views of the causes, sexes, ages, duration, periods, &c. of which we can take but a cursory notice. Thus of 116 cases§ of arachnitis, 21 were from percussion, two from insolation, two from tubercles of the brain, ten from depressing passions, six from metastasis, one from hydrophobia, three from apoplectic tendency, seventeen from indirect causes, 54 from unknown causes. Of these 116 cases, 78 were suppurated, and 38 not suppurated. In respect to sex, there were 88 cases of arachnitis in males to 28 in females. By the table of ages it appears that 29 were

---

* That layer reflected over the cerebrum, as distinguished from that lining the dura mater. Rev.

† We this day (26th Oct. 1821) saw a very interesting dissection by Mr. Brodie, of a woman who died of well-marked arachnitis at St. George's Hospital. This was caused also by percussion, and Mr. Brodie pronounced it arachnitis, from the symptoms, and before dissection. There was much coma, and the arachnoid covering the base of the brain was thickened, quite opake, and in some places suppurated. There was scarcely any other lesion of the contents of the cranium besides the inflammation and suppuration of the arachnoid. Rev.

‡ Our readers will remember the sentiments of Dr. Golis on this subject. See No. 7. p. 473.

§ Our authors watched a far greater number than this, but have not thought it necessary to detail more than the above number. Rev.

under 14 years of age—44 between the age of 15 and 30 years—38 between 31 and 60 years of age—five between 61 and 80. The ordinary duration of arachnitis is from seven to eighteen days. Death, however, sometimes happens on the third or fourth day, and patients rarely pass the twenty-fifth. Three cases only were protracted to the thirtieth day.

The course of arachnitis presented to our indefatigable and zealous observers three periods sufficiently characterized by their peculiar order of symptoms.

The *first stage*, or period of increment, is marked by an exaltation of the sensibility, whence proceeds cephalalgia, one of the most constant characters of the disease. A tendency to sopor is sometimes manifested, especially when the arachnitis is seated at the base of the brain. The stomach also is sympathetically affected with nausea or vomiting. A febrile movement is generally established in the system, varying according to the age of the subject, the sensibility of the constitution, and the degree of the inflammation. In some rare cases, especially of metastasis, coma sets in from the beginning, and all the symptoms of the third period or stage (described hereafter) commence at once, and are quickly followed by death. The duration of this first stage is usually from a few hours to three or four days.

The *second* stage or period, which is that of reaction, is accompanied with disturbance in the locomotive powers, corresponding with that of the brain itself. It is in this stage that we observe convulsions, delirium, restlessness, oscillations or commencing dilatation of the pupils, and other phenomena of cerebral inflammation. In this stage cephalalgia is less constant than in the first stage, the sensorium appearing less sensible of impressions, as well internal as external. This stage varies in duration, from two, three, or four days, to one or even two weeks. It exhibits some difference in symptoms according to the principal seat of the arachnitis. When the latter is at the base of the brain or in the ventricles, coma is almost essential, and is combined with convulsions, agitation, affection of the eyes, &c. whereas, if the arachnitis be on the convexity of the hemispheres, *delirium* is the early and regular characteristic phenomenon.

The *third* stage is that of the shortest duration, varying from a few hours to three or four days, and rarely passing that period. This is the stage of collapse;—the abolition of sense, loss of motive power, paralysis, local or general, and coma, being the characteristic symptoms. In this stage, however, the features of various cerebral affections are combined, and consequently all distinctions between arachnitis and inflammations of other parts of the brain confounded.

The *collapse*, so characteristic of the third period or stage, and the *excitement* which distinguishes the second, have this peculiarity, that while one part of the body shall present the phenomena of one of these stages, another part shall present those of the other. For example, in the face, we shall often see the muscles of the eye-lids paralytic, while those about the mouth are convulsed. It is principally when the arachnitis exists about the base of the brain, near the decussation of the optic nerves, that this medley of symptoms belonging to two different stages is observed.

It is almost needless to say, that a return to health from any of these stages (this is rarely the case from the third) is marked by a diminution of intensity in the symptoms, and a final cessation of them. Our authors confess, at the same time, that it is often difficult to distinguish the transition from one stage into another—especially of the first into the second, and the second into the third. No single symptom can be depended on for this discrimination—the whole must be taken in connexion. Six cases are next detailed by our authors, illustrative of the foregoing observations; one or two of which he shall condense for the use of our readers.

" *Case* 1. *Arachnitis of the Base of the Brain.* A child, five years of age, ricketty from birth, but otherwise pretty healthy, was seized, without any known cause, at 2 o'clock, P. M. with violent vomiting, accompanied with head-ache so intense as to cause the child to utter the most piercing cries. This, which was the *first* stage, lasted till midnight, when the child became insensible, uttered sharp cries, and shewed convulsive movements in the arms and eyes, with commencing trismus.

" Next day (second day and period of the disease) the child was brought to the hospital in a deplorable condition. The convulsive motions of the upper extremities were frequent, while the lower limbs were rigidly extended. Sensibility was almost abolished, pupils dilated and almost immobile. Four leeches to the sides of the neck—blister to the nape—sinapisms to the feet—æthereal potion (!) "Infusion de tilleul avec quelques gouttes de liqueur d'Hoffman." In the evening a purgative lavement. In the course of this day, whether by the efforts of Nature or Art, the convulsions ceased, but the vomitings were renewed twice. The other symptoms remained the same. Towards night all the phenomena assumed an increase of intensity.

" The third day was distinguished by the third stage of the disease. The respiration was now stertorous—the pulse nearly imperceptible—coma profound—gradual extinction of all the functions—death in forty-eight hours from the invasion.

" *Necrotomy.* A portion of arachnoid at the base of the brain, and extending in a triangular form backwards to the cerebellum, was inflamed. The brain itself, under the middle ventricle, of a dark colour from blood (sablé de sang)—all the viscera perfectly sound." P. 29.

*Case* II. In the *second* case related, the *third* stage supervened on the *first*, without any second stage. A girl eight years of age, was seized on the 15th Sept. 1820, with a slight febrile movement and moderate cephalalgia. On the second day the fever and head-ache continued. Nothing but the infusion of tilleul (linden tree) administered. On the third day there was, in addition to the other symptoms, some degree of somnolency. The fourth day, in the morning, same state, but quite sensible and rational. In two hours after the visit, she was seized with convulsions of the upper extremities and insensibility. *Evening*, the face red, eyes turned upwards, eye-lids wide open, pupils dilated, grinding of the teeth, foaming at the mouth, coma profound, trismus, pulse frequent, respiration laborious, great heat. Leeches, sinapisms, blisters, moderated the convulsions, but did not arrest the progress of the disease. She died on the sixth morning. The other four cases, one of which is from Morgagni, we must pass over.

*Mode of Invasion*. On the prompt diagnosis of arachnitis all our hope of success in the treatment must depend. The mode of invasion of arachnitis would be easy of detection were it always to take place in a previously healthy subject, and uncomplicated with other acute or chronic affection. But these complications or superventions are of frequent occurrence, and too often throw the practitioner quite off his guard, at the commencement of this dangerous malady. Our authors attach (and justly too) the greatest importance to a discrimination of the early diagnostic symptoms; and the reader must therefore have patience, if there appears a tedious minuteness, or occasional tautology in the descriptive parts of this treatise. Let him remember the words of the great philosopher—"Nihil est aliud magnum quam multa minuta." Let him also bear in mind that his task of *perusal* is easy, when compared with ours of analysing, translating, and condensing the original.*

---

* The powers and advantages of the press are prodigious, and only fail to excite our astonishment as well as admiration, by being so familiar to us. A single example, and that of a comparatively insignificant kind, will illustrate this remark:—thus the literary labour of an individual, constructing this article, can be commanded by any person, in the remotest part of Europe, or of the world, for the sum of about as many pence as it cost days or rather nights in compiling! Intellectual labour would bear a very different price were it not for the invention of types.

"The symptom of the greatest importance, as was before observed, in the invasion of arachnitis, is *cephalalgia.* It is often intense, but varies considerably according to its seat. More than two-thirds of the patients presented this symptom, and probably all would have done so, had they been seen at the very commencement, or had they been capable of giving a faithful account afterwards, when brought to hospital. Cephalalgia then, occurring suddenly, and especially when violent, should always excite suspicion of arachnitis, whether it takes place in a person previously well, or labouring under some other disease. This suspicion would be strengthened by the occurrence of any disorder of the intellect, the organs of sense, or the loco-motive powers. It is necessary, of course, if an external injury be inflicted, to bear in mind that the pain *may* be from inflammation of the scalp or pericranium; but where there is any doubt about the matter, it is safest to consider the seat of inflammation as internal rather than external of the cranium.

"After cephalalgia, in point of importance, comes disturbance of the intellectual faculties, varying in form, according to the habitudes of the patient. It is never very violent from the beginning. Disturbance of the digestive organs (sympathetically) is particularly conspicuous in the approach of arachnitis—indicated by nausea, or even vomiting, especially after taking any food or drink. Disturbance of the muscular system, at this early period, is a rare occurrence, and is generally *partial,* consisting of convulsive twitchings or motions of the upper extremities or face. When this symptom presents, we may generally conclude, that the period of *invasion* is past, and that arachnitis has been going on for some time unobserved."

Lastly, we remark derangement of the organs of sense, such as tinnitus aurium, errors of vision, faintness, vertigo, strabismus, change of features. These last three symptoms are often the only cerebral ones which mark the onset of arachnitis in children, especially when occurring at the base of the brain. On this account, in such subjects, we are to examine if there be any attendant pyrexia—(a phenomenon which is little to be relied on in other circumstances)—redness of the conjunctiva, flushing of the cheeks, and other symptoms indicative of cerebral congestion. Coma, in the invasion of arachnitis, is exceedingly rare.

In the beginning of phlogosis of the arachnoid, from an external cause, we may sometimes remark a disposition to rigor, accompanied by a dryness of the wound. But, in general, these rigors are indications that the suppurative process has commenced in the membrane.

*More particular Analysis of Symptoms.* Impressed with the vast importance of being able to detect, and that at an early period, so dangerous a malady as arachnitis, our authors do not content themselves, as most medical writers do, with a mere *enumeration* of symptoms. They go into a particular analysis of each class, chusing rather to run the risk of being accounted tiresomely minute or tautological, than defective in the history and delineation of the disease which they have undertaken to pourtray. It is very probable that the reader (especially he who prefers a theoretical discussion to a dry detail of facts) may be somewhat fatigued with the first perusal of these delineations; but when a case occurs (and often will it occur) wherein the life of the patient and the reputation of the practitioner are at stake, he will turn, with trembling anxiety to these pages for assistance, and then he will think us any thing but prolix. The great utility of books, indeed, is useful reference in time of need.

" *Face or Countenance.* Every observant practitioner knows how important it is to study the expression of the countenance in diseases. In arachnitis, besides a general character of surprize and stupor, (etonnement et stupeur) which it is impossible to describe, but which cannot easily be mistaken, after being once seen, the most prominent symptoms are furnished by the eye. The pupils are dilated or contracted, or alternately in each state. The globe of the eye presents a greater or less degree of redness in the conjunctiva—squinting on one or both sides—constant rolling of the organ—its reversion upwards—and finally paralysis of the upper eye-lid. In more than a third of all the cases, affection of the pupils was observed. Absolute immobility of pupil does not take place till towards the close of the third stage. Redness of the conjunctiva is very frequent, often amounting to actual ophthalmia. Strabismus occurred in about a tenth of the cases, and, together with the rotation of the eyes, generally was seen towards the third stage. Turning up of the eyes, and paralysis of the eye-lid, were much more frequent than strabismus. They usually occurred towards the end of the second, and in the third stage. Errors of vision are not of much consequence, but morbid sensibility of the retina to light is a striking feature of arachnitis. All the ocular phenomena, however, are more conspicuous when the arachnoid of the base of the brain is affected, than when that of the convexity is inflamed."

The muscular actions of the face are often greatly deranged, and ought to be carefully noticed. Trismus is by no means a very unfrequent attendant on arachnitis, whether suppurated or not. Full a fifth of the cases presented this symptom. It seldom occurs till after the first stage is past. Grinding of the teeth, and foaming at the mouth, are generally seen only among children; and in the second and third stages of the disease. Spasmodic or convulsive movements of the facial muscles are not very frequent, and never seen but in the advanced stages. Generally speaking, the face is coloured and animated in arachnitis—sometimes, however, our authors found it pale and expressionless.

*Sensitive Apparatus.* This apparatus furnishes symptoms the most frequent of occurrence, the most easy of detection, and the most characteristic of the disease, under the form of disturbance of the intellect, as delirium, somnolency, coma, cephalalgia, stupor;—and, finally, derangement of the general sensibility, and of the senses in particular. Delirium more frequently affects adults, who are most disposed to arachnitis of the convexity of the brain. It is generally of the tranquil kind, or a muttering of half-articulated words between the teeth. The delirium is not usually so intense but that the patient can be roused to answer distinctly at times. These remarks appertain to the first and second stages. In the third, there is generally annihilation of the intellectual faculties. The commencement of delirium may, for the most part, be considered as the sign of transition from the first to the second stage, and forms the most characteristic feature of arachnitis of the convexity of the hemispheres. Where there is no delirium, there is generally either dulness, moroseness, irascibility, or preternatural excitement, and unusual exhilaration. In almost all cases, however, we see a marked diminution of the cerebral faculties, or an impossibility, as it were, of bringing them into action—so much so, that many patients can only be induced to utter monosyllables.

Somnolency (assoupissement) is one of the most frequent of all the phenomena of arachnitis, having been observed by our authors in 82 out of 116 cases detailed in the volume before us. When arachnitis pursues its ordinary course, this symptom does not appear till the end of the first or beginning of the second stage. In a very few cases our authors observed this somnolency from the very commencement of the disease. 55.

Of *cephalalgia* a good deal has already been said. It is almost inseparable from the first and part of the second stage. It is highly probable that it continues to the last, though not complained of by the patients, who are overwhelmed by the force of the disease, and incapable of distinguishing any particular symptom. They generally characterize the pain as heavy, numb, or shooting—usually occupying the whole, but sometimes only half of the head. The apparent seat of the pain is not always the seat of the disease, as will be shewn hereafter.

*Stupor,* characterized by a kind of self-abandonment, loss of all energy, and countenance of surprize, is common to every stage of the disease, but especially the two first. In a very few cases there has been obstinate pervigilium, instead of somnolency—and in a still fewer, (shewing that there is no rule without exception) the patients have preserved the integrity of their intellectual functions till the last.

The muscular, or loco-motive system exhibits various deviations from the healthy state, viz. general or local rigidity, or contraction—local or general palsy—local or general convulsions—to which we may add a third state, that of agitation. This last symptom was observed in about a fifth of the patients, and only in the first and second stages. It is not of much importance as an aid in the diagnosis—but not so *convulsions*, These, with paralysis, are one of the most characteristic signs of arachnitis. General convulsions were observed in one third of the whole number. They are most common in children; and are principally seen in the second and beginning of the third stage;—more pronounced in the upper than in the lower extremities. Rigid contractions of the muscles are seen (in order of frequency) in those of the lower jaw, posterior of the neck, superior and inferior extremities. They belong to the second stage, and early part of the third—sometimes constant, but generally shewing intervals of relaxation. These contractions are not so rigid but that they may be overcome by a steady resistance of the hand of the by-standers. Our authors have several times seen *hemiplegia* suddenly supervene in the course of the first and second stages of arachnitis—generally when the inflammation was caused by external violence. 60.

The disturbances in the *digestive organs* have been sufficiently noticed under the head of invasion of arachnitis; and as to the state of the *vascular system*, pyrexia is so common an attendant on all the phlegmasiæ, that it can give us no aid in the diagnosis of arachnitis. The organs of respiration offer nothing that can much elucidate the diagnosis in question.

The temperature of the skin is generally elevated, and equally diffused over the whole surface of the body, being highest in the second stage of the disease, diminishing and greatly varying towards the termination of the third stage. The skin is generally dry during the first stage—in the second, sometimes moist, or even covered with an abundant perspiration—especially about the face. The decubitus offers nothing particular. It is the same as in all other diseases affecting profoundly the animal economy—particularly where the brain and its functions are much compromised. The patient lies prostrated, as it were, on his back, deprived of all energy, and every member and part of the body taking position according to the laws of gravitation. This decubitus only applies to the end of the second stage and the whole of the third. Our authors here take notice of a disagreeable odour which patients labouring under this disease, exhale about the end of the second stage, and which they can compare to nothing but the smell of mice. It always proved a very unfavourable symptom.

*Mode of Termination*. Few, comparatively, recover who exhibit unequivocal symptoms of arachnitis. Nevertheless, in a considerable number of patients who had died of other diseases, and who had previously laboured under inflammation of the arachnoid, our authors found unquestionable traces of this peculiar phlogosis, such as adhesions and thickenings, proving that arachnitis is occasionally susceptible of cure. Farther on will be stated the mode of transition from the acute to the chronic form of the disease—a transition, in the experience of our authors and others, very frequently determining mental alienation. It is obvious that the prognosis in this inflammation is generally most unfavourable. Profound somnolency announces that the patient has but three or four days to live, and nothing but a sensible and progressive diminution of the symptoms ought to induce us to offer a favourable prognosis.

III. *Pathological Anatomy*. The various organic lesions discoverable on dissection of patients who have died of arachnitis are, 1st. a simple blush or redness of the arachnoid membrane. 2d. Thickening, augmented density, and loss of transparency in the said membrane. 3d. A purulent, sero-purulent, or sero-gelatinous exudation on its surface. 4th. The formation of false membranes. 5th. A serous effusion into the ventricles, between the laminæ of the arachnoid, or into the cellular tissue which unites the said membrane to the pia mater.

"The *redness* is generally confined to some spots on the convexity and base of the brain—sometimes, however, extending over a whole hemisphere, and penetrating even into the ventricles; but this last circumstance is very rare. This redness varies from a slight rosy tint to that of the deepest scarlet;—and should be distinguished from simple congestion of that membrane, as well as from injection of the vessels of the pia mater beneath. In the latter cases the membrane may be raised with a scalpel, and will then appear transparent —not so in the inflammation of the tissue.

"Often, instead of this redness, we find a *thickening* and *opacity* of the membrane. It may then be said that inflammation has disorganized its structure, in the same way that opacity is produced in the transparent cornea by violent ophthalmia. This thickening is seldom general; but distributed in stripes or patches more or less extensive, and always thicker and denser in the middle than at the borders." 70.

*Suppuration* of the arachnoid is a frequent termination of the disease, and presents some difference of aspect. In most cases the pus is diffused over the surface of the arachnoid, forming a very thin layer, little adherent to the membrane, from which it may be easily scraped with a scalpel, the membrane then appearing thickened, red, and sometimes slightly villous. It is but rare that the pus is found collected in a focus in any considerable quantity. It varies a good deal in colour, nature, and consistence; and sometimes, instead of pus, we find only a serous infiltration into the cells of the arachnoid, resembling much the œdema of the glottis, after acute inflammation of that part. This purulent secretion is most commonly found at the convexity of one or both hemispheres, er else on some points at the base of the brain—particularly about the decussation of the optic nerves, and the tuber annulare. Another kind of effusion is a gelatinous layer, very much resembling the substance found in certain encysted ovarian tumours. On squeezing this layer a liquid is forced out, and a kind of unequal membrane is left between the fingers. This peculiar infiltration is usually found at the base of the brain, near the optics or pons varolii.

"At other times the arachnoid is covered with a complete *false membrane*, similar to that found on the serous tissues in other parts of the body. Its organization was sometimes so far advanced as to exhibit vascularity. These false membranes were more frequently found on the convexity of the cerebrum and cerebellum, than at the base of the brain, or in the ventricles," 72.

*Serous Effusion* was found in almost every case of arachnitis; but *generally* not exceeding an ounce in quantity—often, however, amounting to three, four, or six ounces. This liquid is *usually* contained in one, or in both of the lateral ventricles—sometimes, however, there is effusion in all four ventricles—and frequently it is dispersed over the whole surface of the arachnoid, which then appears bathed therein. This serosity accumulates at the base of the skull when we remove the brain. It is almost always limpid, occasionally milky, or even flocculent. This serous effusion is particularly apt to occur in arachnitis of the base of the brain, and of the ventricles. It is also more frequent in children than in adults.* When *adhesions* of the arachnoid present a cellular appearance, it is a proof that the arachnitis is of old standing. These adhesions, in Bichat's experience, degenerate occasionally into bone. Slight granulations on the arachnoid may sometimes be seen, by means of a good light, and appear to be of the same nature as those seen in the pleura and peritoneum after inflammation of those membranes.

* Our authors, of course, take care not to confound the effusion of arachnitis, with the dropsical effusions seen in the brains of old people.

IV. *Pathological Physiology.* This division of the work comprises an attempt to trace the connexion of outward signs with inward changes—to examine whether this connexion be subjected to any thing like regular laws, or varies with every idiosyncrasy of constitution, and thus becomes entirely personal or individual. Faithful to their plan, our authors appeal solely to facts, and decline all theory. The only plan of effecting the object of this division is, of course, a careful comparison of symptoms during life, with appearances after death. Of this comparison we now proceed to convey some account to our readers.

" *Dilated pupils* cannot be considered as proof of effusion into the ventricles, or between the coverings, or at the base of the brain; and for the following reasons:—of 26 cases with dilated pupils, eleven presented effusion into both ventricles—two into one ventricle—four with no dropsy of the ventricles, but effusion on the surface and at the base of the brain—one with effusion on the surface of one hemisphere only—eight without any trace of effusion in any part of the brain." 82.

From this statement it is evident that we cannot place implicit reliance on the phenomenon of dilated pupil in cerebral affections. Indeed, we have been so often disappointed by this symptom, that we have long ceased to attribute much importance to it, unless in connexion with various other phenomena. It certainly is *then* of some consequence.

" *Case* III. Desforges (Mary) aged 38 years, was brought to the Hotel Dieu, on the 15th July, 1818. Only the following short history could be obtained. She had experienced, for a long time, anxiety, malaise, lassitude, disinclination for all kinds of exercise, wandering pains in the limbs and back, constant and severe headache. These symptoms daily augmented; and after the imprudent administration of an emetic, the cephalalgia in particular, became so exasperated as to produce delirium, with convulsive movements in the extremities. This state had continued three days, when she was admitted into the hospital, presenting the following symptoms, (on the day of entry she had been bled twice,) viz. face pallid, tongue moist, abdomen soft, but painful on pressure, particularly the epigastrium and right iliac region, respiration impeded, stertorous, and more frequent than natural, chest sonorous at all points, pulse slow, full, strong and regular, spasmodic contractions of the upper extremities, subsultus tendinum, eyes watering, conjunctivæ red and injected, *right pupil contracted, left dilated,* both immoveable, squinting (outwards) of both eyes, profound somnolency, constant moaning, eye-lids half closed, temperature slightly elevated, covered with cold clammy sweats. Twelve leeches to each side of the neck, and twelve on the abdomen. Died in the night.

" *Dissection.* The whole of the arachnoid covering the upper and lateral surfaces of the hemispheres thickened, opake, and denser than natural. A very thick puriform layer was uniformly diffused between the arachnoid and subjacent pia mater, while the same kind of fluid was infiltrated between the laminæ of the former membrane. There was no effusion into the ventricles, and the arachnoid lining them was healthy. The brain itself and cerebellum were perfectly sound; nor was there any disease in the abdominal or thoracic viscera." 87.

*Contraction of the Pupils.* There is the same uncertainty attached to this as to dilatation. Nine cases presented this phenomenon in both eyes. In six of them there was serous effusion in both ventricles—while in the other three there was no trace of effusion any where in the brain.

"*Case* IV. Hubert Francois, 25 years of age, of feeble and delicate constitution, was received into the Hotel Dieu, in the month of March, 1814, for a catarrhal affection, to which much attention was not paid. Three weeks after his entrance, as he continued weak and without appetite, he was put on a course of bark and bitters, which, after two days trial, were laid aside, as restlessness, watching, spasmodic movements, and afterwards a kind of somnolency supervened. These symptoms went on increasing the succeeding day;—and, in the morning following that, there was loss of sensibility, with complete immobility. The pupils were remarkably contracted, especially the left—the breathing was embarrassed—the pulse became feeble, and death closed the scene on that evening.

*Necrotomy.* Two ounces of limpid serum in the lateral ventricles. The arachnoid covering the whole of the left hemisphere thickened and opake, with an albuminous concretion of more than a line in thickness beneath. In the fossæ occipitales of the left side the arachnoid was evidently inflamed and covered with an albuminous concretion, though less consistent than that on the left hemisphere. The bronchia were somewhat reddened. All the other viscera of the thorax and abdomen were perfectly sound." 92.

*Rotation of the Eye.* This was only seen in five cases, and in all of these there was suppuration of the arachnoid about the tuber annulare, and in four of the five, effusion also in both ventricles. Although no positive indication can be drawn from five cases, yet rotation of the eye-balls may fairly be regarded as a dangerous symptom.

*Strabismus.* This was often found to attend suppuration of the arachnoid, especially about the decussation of the optics; but no dependance can be placed on the phenomenon, except in conjunction with various other symptoms.

*Trismus* was a symptom occurring in the different lesions attending arachnitis, and therefore not peculiar to any.

*Distortion* of the mouth is a very frequent symptom of lesions affecting the cerebral pulp itself, as our authors will shew in a future work; but in arachnitis it is not to be depended on.

*Coma*, which, in affections of the brain itself, generally indicates pressure from effusion or extravation, is far from being a certain indication of such pressure in arachnitis. It was often seen by our authors where there was no effusion or suppuration, but simply inflammation of the arachnoid, especially about the basis cerebri, decussation of the optic nerves and tuber annulare. On the other hand, there has been no coma where the arachnoid has been suppurated, and sero-purulent effusion pressing on the brain. We shall introduce a short case in illustration.

" *Case* V. On the 1st April, 1814, a man, 60 years of age, plethoric, athletic, and of apoplectic constitution, was brought to the Hotel Dieu. He had been three days ill, occasioned by a violent fright, and presented the following symptoms:—face flushed and animated, nictitation of the eye-lids, convulsive twitchings of the facial muscles of the lower jaw, and of the lips, confusion of intellect, calling for people absent, reviling those present, believing himself falling into water, over precipices, &c. and using violent exertions to avoid imaginary perils. All the limbs were in constant convulsive motion. This state continued the whole of the day, and he died that night.

*Dissection.* Under the dura mater was found a kind of clot formed by a thickening of the arachnoid covering the right hemisphere from infiltration of a sero-purulent fluid. The rest of the arachnoid, and the brain itself perfectly sound, as were all the other viscera." 103.

*Cephalalgia.* This accompanies all lesions of the arachnoid, and has been amply noticed in the symptomatology.

*Delirium.* This seems particularly connected with inflammation (previous to suppuration) of the arachnoid covering the convexity of the cerebrum or cerebellum. It is oftener observed in young people, and in people where there is strong reaction; rarely or never seen in arachnitis of the base of the brain.

*Hemiplegia.* In eight cases of this affection our authors found effusion on the convexity of the *opposite* hemispheres. This phenomenon was observed principally in arachnitis from external violence.

*Convulsions*, *Spasms*, *Rigidity*. These are generally on the *same side* as the inflammation or effusion; but no certain indication can be drawn from their existence or non-existence. In conjunction with other phenomena, they render the prognosis more unfavourable.

*Vomiting*, as was said before, is a very constant attendant on arachnitis, from the intimate sympathy existing between the brain and stomach; but there is no positive indication to be drawn from this phenomenon, as to the part of the arachnoid affected, or the exact state of the lesion, whether it be inflammation, effusion, or suppuration.

V. *Treatment.* Although arachnitis is a most dangerous and fatal disease; yet there can be no doubt that it may be frequently arrested in its destructive progress by judicious treatment, if taken in the early stage. If once entered on the second stage, recovery will be rare—our French brethren say impossible; but then the comparative inertness of their practice may account for their constant failure after the first stage of the disease is past.

*Bleeding.* This holds the first rank in the list of remedies, of course. It must be from a large orifice, so that the rapid effusion of blood may induce syncope, or an approach to that state. Our authors have had repeated opportunities of witnessing the good effects of depletion carried to this extent, which dissipated, as by a charm, the most excruciating head-aches, and other phenomena attendant on, or ushering in, arachnitis. They recommend the opening of two veins at once to effect this purpose more speedily and more powerfully. This is a bold step for a French physician, and smacks not a little of that energetic British practice which it is too much the fashion in France to deride as empirical, and designate as Herculean.

In respect to the part from which blood should be taken in preference, our authors are rather undecided. It so happened, they observe, that it was from the arm the majority of the patients were bled, and though venesection, in this way, was often successful; yet, from a few other cases which were more particularly under their own immediate superintendance, and where the blood was drawn from the lower extremities, they thought the effect was more prompt and decisive in this than in the former mode. It must be exceedingly difficult to draw just comparisons of this kind; and though we by no means hold in contempt the doctrine of revulsion, as is too generally done in the present day, yet we cannot see the superior advantage which is likely to ensue from bleeding in the feet, in cases of cerebral inflammation, especially when we know that in all other inflammations, the nearer the bleeding is to the seat of disease, the better. In inflammation of an internal and vital organ we venture to assert that the venesection which is freest and most readily performed is the best; and, in this respect, brachial has the advantage over tibial or pedal bleeding.

Our authors have not been able to appreciate the effects of temporal arteriotomy or bleeding from the jugular vein, on account of the paucity of such operations, and the *difficulties* (as they state) which attend their performance.

They properly observe that the quantity of blood to be drawn, and the repetitions of blood-letting, must depend on the age and constitution of the patient, together with the intensity of the disease. They are probably right also in asserting that bleeding can be of no service except in the first stage, or in the very first moments of the second. Nevertheless, should symptoms of excitement or plethora occur in later stages of the disease, blood should be abstracted on the principle of euthanasia or lessening the sufferings of the patient, even where we have no hope of averting the fatal issue. We think this advice judicious in almost all other diseases as well as arachnitis, and for another reason that our authors have not given—namely, the *uncertainty* of all medical events, diagnostic and prognostic, and consequently the *chances*, however feeble, that often exist in circumstances apparently the most desperate.

*Leeching*. Although general bleeding forms the basis of the treatment in this and all other dangerous inflammations, yet local abstractions of blood, especially by leeches, offer very important resources and assistance to the principal measure. This local blood-letting is peculiarly useful in children and in debilitated subjects, especially after general bleeding has been carried as far as we dare venture to go. In arachnitis the places of application are usually the sides of the neck, the temples, the nape, and behind the ears. In the case of young children, four or six leeches behind each ear, will give issue to a sufficient quantity of blood at a time; but to those of ten or twelve years of age, eight or ten leeches may be applied on each side, and so on according as the age advances to manhood. As in general blood-letting, they think the leeching should be carried to syncope in the early stage of the disease. In all cases where the patient is at all robust, our authors believe the local application of leeches do more harm than good, unless one or two copious general bleedings have preceded them. Our authors have seen the most beneficial effects result from the application of a cluster of leeches to the crown of the head in cases of arachnitis, especially where there was reason to believe, from the symptoms, that the inflammation was on the convexity of the hemispheres. M. Recamier, a physician said to be rich in therapeutical expedients, is in the habit of applying leeches in this way. They have not drawn blood from the scalp by scarifications, and think leeching superior. But cupping the temples, back of the neck, or between the shoulders, they conceive to be a valuable remedy, though too much neglected in France.

Immediately after blood-letting, or even during that operation, the feet and legs should be immersed in warm water, rendered more stimulating by the addition of mustard, salt, or muriatic acid. They should be kept immersed in this fluid half or three quarters of an hour. The bath should be hot enough to redden the skin, and draw the blood forcibly to the lower extremities.

*Sinapisms* have a still more powerful effect than the pediluvia; but our authors very properly remark that, in many constitutions, they produce a degree of re-action or excitement in the system, which has the effect of increasing rather than diminishing the cerebral congestion or inflammation, especially if copious sanguineous depletion have not preceded. They ought, in general, to be removed as soon as the skin is well reddened, or what is better, our authors think, a piece of fine muslin should be interposed between the sinapism and the skin. This prevents all the mischief, and detracts nothing from the efficacy of the remedy. Our authors condemn the application of sinapisms to the soles of the feet, as preventing the subsequent use of pediluvia. The inside of the legs and the knees are the best places.

*Blisters.* Of these our authors cannot speak with much confidence or certainty. As they have seen some cases, however, where violent cerebral affections were quickly dissipated by blisters covering the whole of the shaven scalp, they think this measure should be borne in mind as a useful auxiliary in these dangerous cases. At the same time they propose, as a still more energetic and sudden counter-irritant, the ammoniacal pomatum, lately recommended by Dr. Gondret.

*Purgatives.* Our authors very properly remark that, in a malady so dangerous as arachnitis, there is no part of the body to which we should not, by counter-irritants, endeavour to draw the irritability of the system, so as to prevent its concentration around the part inflamed. On this account, we should not neglect purgatives, whose effects on the intestinal canal are analogous to those produced on the skin by blisters, &c. Our authors seem to forget that another, and perhaps the most important effect of purgatives, is to reduce the quantity of circulating fluids in the body, by the rapid increase of secretion which they produce on the mucous membrane of the primæ viæ, and thus aiding sanguineous depletion in a very powerful manner. They recommend, as purgatives, calomel, rhamnus catharticus, castor oil, or salts, combined occasionally with the drastic resinous purgatives. Desault and Bichat were in the habit of prescribing large doses of antimony or ipecacuan, in glysters, when inflammation supervened on wounds of the head :—and this measure our authors recommeud in arachnitis, though their own experience is is not sufficient to afford positive data on this point.

Our authors do not mention, and seem not to be aware, that there are certain medicines which, taken internally, exert considerable control over the action of the heart and vascular system, such as digitalis and tartrite of antimony. In this country these remedies are productive of considerable effect as aids to depletion by the lancet and purgatives.

*Local Application of Cold.* At the first glance, it might seem that the application of ice and of blisters to the scalp, involved some degree of incongruity. But the object—indeed the effect of blisters is to draw an afflux of blood and sensibility to the exterior of the head, in order to lessen irritation or inflammation of the interior. The object of cold is to constringe the vessels of the head generally, and lessen the sensibility of its membranes, thereby reducing inflammatory excitement or congestion of the brain or its coverings. Our authors think there is no case of arachnitis, in its first and second stages, which forbid the local application of cold. It is agreeable to the feelings of most patients, and often restores them to their senses when previously delirious, while at the same time it mitigates head-ache.

*Position.* This is of great importance in all diseases of the head. The laws of living do not destroy the laws of dead matter, in all cases. Blood will gravitate to the most depending point of the body, before as well as after death; and therefore in inflammatory affections of the brain or its meninges, the head should be kept as high as possible. It is evident also, that light and noise are to be carefully excluded, in order that no stimulus be directed towards the sensorium through the medium of the nerves of sense.

*Compression of the Carotids.* In the first volume of our *quarterly* series, p. 498 *et seq.* we gave an account of Dr.

Blaud's experiments and observations on compression of the carotid arteries, in cases of sanguineous turgescence of the cerebral vessels. Our authors refer to Dr. Blaud's paper, and quote some of his cases, but have not had experience of the measure themselves.

*Trephine.* Our authors have heard proposed and seen executed, the perforation of the cranium by the trephine, with the view of arresting the progress of arachnitis! They very properly condemn the measure as much more likely to increase than to remedy the evil.

*Affusion of Cold Water.* This is a remedy for arachnitis which is little dreamt of by practitioners in general, but from which our authors have seen such decisive good effects, in numerous cases, that they go into very minute details respecting its mode of application, and its modus agendi, convinced of the great importance of the measure in this formidable disease.

The phenomena attendant on the affusion of cold water over the surface of the body, are well known in this country, since the classical exposition of Dr. Currie appeared. But we believe that neither Dr. Currie nor any of his followers contemplated the application of this powerful remedy in cases of topical inflammation, especially of a vital internal organ. But all reasonings and theories must give place to facts, however ingenious or well constructed the former may be. Dr. Recamier has put this remedy to the test of experience on a very large scale at the Hotel Dieu, of which our authors were witnesses, besides employing it in their own practice. They aver that the primary effects of cold water poured on the head and over the body are directly sedative on the surface, and through sympathy, on the brain and nervous system generally. They have repeatedly, indeed always, seen patients delirious from arachnitis, recover their senses *instanter* on affusion being employed—this amelioration of symptoms being of longer or shorter duration according to the intensity of the disease. In the reaction which succeeds the cold affusion, we might naturally expect an exasperation of the topical inflammation; but experience proved it to be otherwise; for as the heat gradually expanded itself from the centre to the circumference of the body of the patient, the restlessness diminished or ceased—the pulse became full but not frequent—the thirst decreased—the intellectual faculties recovered more energy—the functions of the secreting organs were restored—the respiration became free, and a kind of temporary crisis is established. It is at this period that the patient labouring under arachnitis experiences the greatest degree of benefit from the cold affusion.

"Cet ebranlement général, opere sur le systeme nerveux, conjointement avec le dégorgement sanguin des meninges qui resulte de l'application directe du froid sur la tête peuvent amener et amenent quelquefois, en effet, une revolution favorable à laquelle beaucoup de malades ont dû leur salut." 145.

This amelioration of the disease resulting from the cold affusion generally lasts several hours, at the end of which time the cerebral phenomena and all the pyrexial symptoms return, sometimes running higher even than before the affusion, but at others, ending in a state favourable to a return of the healthy order of things. If a collapse of the system succeeds, the prognostic is most unfavourable.

Our authors think that the end of the first and beginning of the second stages are the best periods, by far, for the employment of the cold affusion. At these periods the patient has strength enough to produce a sufficient degree of reaction. When the third stage has taken place, the organic changes in the inflamed tissues preclude almost all chance of recovery; and the cold affusion, of course, must be unavailing. Nevertheless, as "*melius anceps quam nullum remedium*," they think in these desperate circumstances the affusion should be tried as a forlorn hope. Our readers will easily perceive, that in all cases where this measure is employed, the rules and precautions laid down by Dr. Currie in its application to fevers, are to be attended to carefully. The mode of administration is also precisely the same as in fevers. Our authors very properly prohibit the cold affusion in cases where arachnitis is complicated with inflammation of the other viscera, but especially the thoracic organs. They conclude this part of the work with some judicious observations on the management of convalescence. As the brain is the organ of thought as well as the centre of sensation, it is essentially necessary to guard the convalescent from all moral emotions of a disagreeable nature.

VI. *Clinical Illustrations*. We now come to the last and most important division of the work, the illustration of principles by numerous cases and dissections. We must not pass over these slightly—at the same time, we shall endeavour to select those instances which convey the most useful lessons, and pourtray them with all the analytical brevity of which our language is capable. We may remark here that our authors have arranged the cases according to the supposed causes producing them, beginning with arachnitis from *external violence*, the most frequent cause of all. We may also observe, en passant, that the present article deserves perusal from the *surgeon* as well as the physician, since injuries of the head form a very important class of accidents, coming particularly under the cognizance of the former. To the general practitioner these cases are, of course, sufficiently interesting, and we hope will be found, in no mean degree, instructive.

*Case* VI. A bricklayer, 22 years of age, received a contused wound on the left parietal bone by the fall of a mortar trough from a considerable height, which stunned him completely for a short time, when he again recovered his intellectual faculties. He was affected with spontaneous vomiting, and was carried the same day to the HOTEL DIEU, and bled twice on that day. He had also administered nauseating doses of emetic medicine in glysters. All went on well till the *thirteenth* day, when he experienced irregular chills, loss of appetite, and an erysipelatous eruption on the face. This erysipelas ran its course without accident till the 25th day, the wound of the scalp preserving a healthy aspect. On the 26th day, loss of appetite, dryness of the tongue, general uneasiness, diminished suppuration of the wound, some loss of power in the members of the right side betokened no good. These symptoms increased in force during the 27th and 28th day—nights restless—whey for nourishment. 29th. Continual moanings—sensible when spoken to—right arm almost motionless—no discharge from the wound—pulse precipitous.—*Died in the evening.*

*Dissection.* Pericranium detached from the bone and covered with pus—external table of the bone itself, in the site of the wound, necrosed to the size of a shilling, with a fissure of some inches in length. Corresponding to the wound the arachnoid was inflamed some inches in extent—the lamina reflected over the internal surface of the dura mater being considerably thickened, and adherent to the lamina reflected over the pia mater in such a manner as to form a kind of pouch filled with pus. Every other part of the brain and its coverings sound. 173.

This case shews us that an erysipelatous eruption on the face, after wounds of the head, ought to strongly excite suspicion of internal inflammation. It is evident that the arachnitis did not commence here till the 13th day at the earliest, or rather the 26th day, and that the local nature of the mischief might at last have been remedied, in all probability, by the trephine. When the erysipelas appeared, depletion was not put in force; and when things wore a more alarming appearance, no incision was made to examine the state of the bone. We appeal to our brethren, surgical and physical, whether the medical officers of HOTEL DIEU did not "leave undone those things which they ought to have done?" We entreat the attention of our surgical brethren to the following case, on account of the eminence of the practitioner under whom it occurred.

*Case* VII. A man, 30 years of age, was entangled under a pile of falling wood, and was carried, the same day, in a state of insensibility, to the Hotel Dieu, where he was placed under the care of M. Dupuytren. There was a wound, of about an inch in diameter, on the anterior superior part of the left parietal bone, the latter being fractured, and a small portion depressed more than two lines below the level of the rest of the bone. Several incisions were made, and the depressed bone elevated, when the patient immediately became sensible. In the evening of that day there was some cephalalgia, and *eight ounces* (deux palettes) of blood were taken from the arm. The night was good. Next morning there was increase of headache, derangement of ideas, full, strong, and hard pulse. *Emetic whey* was ordered. In the evening the same state continued, *Eight ounces* of blood abstracted. Third day, the ideas incoherent, eyes fixed, face flushed, inclination to throw off the bed clothes. Abstraction of *eight ounces* of blood. Fourth day, incapable of answering questions. At the bottom of the wound the dura mater was seen raised up a little, and covered with pus. Bled from the feet; sinapisms. Temporary diminution of the stupor. In the evening complete insensibility—respiration difficult—cough—dura mater more tense. A small puncture made in that membrane, which gave issue to a spoonful of reddish serum. Thirty leeches to the temples. In the morning of the fifth day there were some trifling signs of returning sensibility; but all things got worse, and death closed the scene on that evening.

*Necrotomy.* Besides fracture, there were some fissures running in different directions. A clot of blood, the size of a six franc piece, was found between the bone and dura mater. The whole of the arachnoid covering the hemispheres was thickened, opake, and covered with pus. The pia mater was also infiltrated. A portion of cerebrum, corresponding to the depression of bone, was ecchymosed, and altered in its colour. The pleura of the right thoracic cavity was inflamed, covered with false membranes, and a considerable effusion of serum on that side." 179.

Our readers will readily perceive that M. Dupuytren, the first surgeon in France, permitted the ravages of meningeal and cerebral inflammation, *after a fractured cranium*, to proceed three days, while he was taking away the dribbling quantities of *eight ounces* of blood, from a man in the prime of life! On the morning of the *second* day, when he found the head-ache increasing, the ideas wandering, *the pulse full, hard, and strong* (pouls grand, plein, et dur) only *eight* ounces of blood were taken, and emetic whey prescribed! No purgatives were given throughout the disease, and swarms of leeches were only applied when the patient was nearly moribaud. If M. Dupuytren's intention was to check cerebral inflammation, we hesitate not to assert that the means he took had not the slightest effect in doing so; and that his practice is condemned by the *precepts* which our authors themselves have laid down, though they make no animadversions on this case of most extraordinary misconduct. This case furnishes also an example of the effects resulting from a rule now almost universally followed in France—never to apply the trephine. We believe few English surgeons would have permitted such symptoms to go on after fracture and depression of bone, without trephining. In this instance there is every probability that the trephine would have liberated the clot of blood pressing on the dura mater, and that active depletion and antiphlogistic measures would have checked the meningeal inflammation. These cases of mal-practice are most valuable, as shewing very unequivocally the dire effects of half measures, when important organs are inflamed.

### *Arachnitis from Insolation; related in Pinel's Nosog. Philos.*

*Case* VIII. "A man, 40 years of age, much addicted to wine, worked hard during a whole day, at hay-making, under a powerful sun. After a frugal repast he went to bed and slept soundly all night. At day break he was found with the following symptoms:—violent head-ache—prostration of strength—involuntary and profuse lachrymation—redness of the face—incoherence of ideas—memory vacillating—extremities cold. This our authors designate the *first stage.* Second day (second stage also) phrenitic delirium—fever. Bled from the feet, antiphlogistic measures. Increase of all the symptoms. *Third day*, convulsions, tremors, death.

"*Dissection.* Inflammation of the dura mater and arachnoid—plexus choroides gorged—slight effusion in the ventricles." 182.

The above is a regular *coup de soleil*, and is not unfrequently seen in the hotter regions of the earth, but is not often so rapid in its march in Europe.

Our authors now proceed from external to internal causes, and first introduce instances of arachnitis from metastasis.

*Case* IX. "A young man, 20 years of age, became affected spontaneously with erysipelas of the face, which exhibited the usual phenomena of that disease. Having exposed himself imprudently to cold, the erysipelas suddenly disappeared, and was succeeded by head-ache, delirium, great restlessness, and convulsive movements of the limbs. Second day (and *third* stage of the disease) profound coma, convulsions, insensibility, unnatural movements of the lips and eyes. This state continued during the *third* day. On the *fourth* day a flaccid state of the members succeeded to convulsions—the breathing became stertorous—and death closed the scene in the evening.

" *Necrotomy.* The arachnoid covering the superior surface of the cerebral hemispheres, was coated with a false membrane, yellow, opake, and very thick towards the sinciput and sides of the brain. The arachnoid covering the upper surface of the cerebellum presented the same appearances. There was also a serous infiltration into the cells of the whole arachnoid, particularly of the left side. The pia mater was injected and red." 186.

We shall not multiply instances of arachnoid inflammation from erysipelas of the face, but proceed to occurrences of the kind from various other causes.

*Case* X. *Arachnitis from suppressed Menstrua.*

" Tissot, a married woman, 27 years of age, having travelled more than 300 miles on foot, experienced a sudden fright, on the 6th October, 1816, producing syncope at the moment. The menses, which were flowing copiously, were instantly suppressed, followed by severe head-ache. On the following day (first stage) there were shiverings, increase of cephalalgia. *Third day*, restlessness, slight delirium. *Fourth day*, very severe pain in the orbital regions—sense of lassitude in the limbs—yellow tongue—frequent pulse. Twenty-five grains of ipecacuan, stimulating pediluvia, barley water and honey. In an hour after the pediluvium, loss of sense, and convulsive movements. Fourth day (second stage of the disease) another accession of loss of sense (perte de connaissance) continuaation of the convulsions, particularly of the right side, trismus, irregular action of the eye-lids, pupils dilated and insensible to the light, face flushed, breathing difficult. Two large blisters to the thighs; antispasmodic potion, demi-lavement. At ten o'clock convulsive movements of the right side, tendency to paralysis of the left. Fifteen leeches to the pudendum, and the same number to the nape of the neck. *Fifth day*, (third stage of the disease,) coma, incomplete paralysis of the left arm, dilated pupil, strabismus, mouth open, deglutition difficult, flaccidity of the limbs, hiccup, intense heat of the skin, pulse strong, frequent, and resisting. Camphor, cinchona, camphorated glysters. Died next morning.

" *Post-mortem Appearances.* Redness of the whole of the arachnoid covering the convexity of the hemispheres, with a whitish purulent secretion spread over the membrane. There was also considerable accumulation of pus, and thickening of the arachnoid at the base of the brain, especially about the decussation of the optic nerves. The membrane lining the ventricles was sound, and only a small quantity of serosity in one of them. The brain and other viscera were sound." 193.

Our authors here relate a case of arachnitis from the poison of a rabid animal. The symptoms of hydrophobia need not be described; but the dissection exactly corresponds with that of a case which we examined ourselves a few years ago. In both cases the arachnoid was found universally thickened and red—the pia mater gorged with blood and serosity—and much serous effusion at the base of the cranium, and in the vertebral canal. There was also inflammation about the cardiac orifice of the stomach, and in the mucous membrane of the small intestines.

Without pretending to explain the nature of the hydrophobic poison, our authors justly observe that by far the greater number of hydrophobic dissections are in correspondence with the foregoing pathological report, and consequently that cerebral inflammation may be expected as a concomitant, though doubted as a cause of rabies canina. We believe that the methodus medendi—if *medendi* shall ever be applied to hydrophobia—must always hinge upon views of this kind.

Our authors are convinced from experience that arachnitis of the base of the brain, and of the lining of the ventricles, is far more frequent in chidren (forming, in fact, hydrocephalus acutus) than in adults. We shall therefore introduce here a few cases illustrative of this too common and too fatal disease.

*Case* XI. " Depuis, 3½ years of age, after enjoying good health, was taken, on the 14th July, 1817, with spontaneous vomiting of greenish matters, which continued more or less till the 24th of the same month, accompanied with prostration of strength, a slight degree of drowsiness, and smart paroxysms of fever, without delirium or restlessness. During all this time there was obstinate constipation, and violent head-ache. The child frequently screamed out, and there were some convulsive movements about the eyes. The intellect did not seem disturbed. He was transported to the Hopital des Enfans, on this day, the 11th of the disease, and the following symptoms were noted. The child lay on his back, the trunk immobile, head turned backwards, eye-lids heavy and closed, pupils little dilated, features altered, eyes turned upwards. The child was sensible when spoken to, and complained of great pain in the occipital region—drowsiness constant, pulse feeble, irregular, and frequent—respiration slow, unequal, and apparently difficult—tongue coated yellow—constipation. Mustard pediluvia—lavements—lemonade—nitric ether mixture. In two hours some convulsive movements—screamings—complaints of violent pain in the back of the head, to which part the child was constantly applying its hand. Twelfth day, (second in hospital,) piercing cries during the whole night—strabismus—features greatly altered. Lemonade, with a grain of emetic tartar—lavements—mustard pediluvia—blister to the nape of the neck and behind the ears—ice to the head. In two hours a strong paroxysm—much crying during the day—loss of intellectual and sentient faculties. *Thirteenth day*, a general remission of all the symptoms—pulse very irregular—great irritability of temper. Same treatment. *Fourteenth day*, (third stage of the disease,)

pupils dilated and immoveable—profound somnolency—pulse irregular, feeble, small, and extremely quick—eyes prominent, affected with strabismus, and half open—head inclining to any position which the laws of gravity dictated—the intellect not much affected. Blisters to the vertebral column. *Fifteenth day*, same state, but the intellect more disturbed. *Sixteenth day*, profound coma—gradual extinction of the vital and natural functions—death.

*Dissection.* Arachnoid inflammation at the base of the brain about the decussation of the optic nerves, and over the tuber annulare—the membrane itself covered, in many places, with an albuminous exudation, penetrating into the fissura magna sylvii. Eight ounces of serous effusion in the ventricles. No other morbid appearance in any part of the body." 237.

We need hardly remark that the *treatment* in this case was admirably adapted to shew the genuine and unsophisticated ravages of disease. Our medicina *perturbatrix* in this country gives us few opportunities, comparatively, of observing these undisturbed morbid processes in perfection. On this account continental pathology is a very valuable article of importation into English medicine. The following case offers a good illustration of what Dr. Golis has denominated the "water-stroke," by the suddenness of the effusion.

*Case* XII. "Petit, 6 years of age, entered the Hopital des Enfans on the 18th March, 1816, without any symptoms of illness. During the 19th also, the child appeared well. On the 20th, in the morning, our authors found him in strong convulsions—tetanic—constant tendency to bend backwards—loss of sense—dilated pupils—difficult deglutition—tension and tenderness of the abdomen—constipation—slow, small, irregular pulse—breathing scarce perceptible—in short, the child was in the *third stage* of arachnitis. 21*st*. General amelioration—incomplete return of sensibility, but continuation of the tetanic rigidity. Sinapisms to the legs—blisters behind the ears—camphorated medicines—ice to the head. 22*d*. Little or no change. The child was revived a little by the ice, but soon relapsed into a state of torpor. Same treatment—two leeches to each side of the neck. 23*d*. Coma regularly established—cerebral congestion well marked—face red—frontal veins swelled—eyes fixed, and turned upwards—considerable rigidity of the limbs—frequent irregular pulse—burning skin—difficult breathing—death in the midst of convulsions.

*Dissection.* Very well marked inflammation of that portion of arachnoid situated between the tuber annulare and the decussation of the optic nerves, in which place the membrane was thickened, and covered with an albuminous concretion. Eight ounces of serous effusion in the ventricles." 238.

We must pass over some hundreds of pages, containing cases and dissections without number, in illustration of every variety and shade of arachnoid inflammation. What we have quoted will, we hope, be sufficient to convey a plain practical view of the attendant phenomena of this dangerous affection. We need hardly wonder that arachnitis should be very generally fatal under the French mode of treatment; yet even *they* have brought forward several unquestionable proofs that the disease is susceptible of cure in many instances, especially when treated with any thing like decision. In this country, we see examples of cerebral inflammation every day subdued by active depletion; and we hope the pathological facts which we have, in this article, laid before our junior brethren, will induce them to pay increased attention to the phenomena attendant on, and indicative of, so destructive and terrible a malady. The following case is a very interesting and satisfactory one.

*Case* XIII. " A man, 45 years of age, of sanguineous temperament, and excellent constitution, and who had served eighteen years in the cavalry, was seized, after a forced march which caused him to perspire freely, with cold chills, head-ache, fever, general uneasiness, (malaise,) tightness of the chest. No medical treatment was employed, and, of course, the man got worse, and at the end of ten days from the invasion, he entered the Hotel Dieu, presenting the following symptoms:—face coloured—conjunctiva injected—pupils immoveable—head-ache—delirium for a few hours past, yet capable of answering correctly when roused—pulse full and quick—skin hot and dry—abdomen painful—respiration impeded—expectoration of a reddish tinge. Sixteen ounces of blood from the arm—leeches to the nape of the neck, to the abdomen, and to the anus. The thoracic and abdominal symptoms subsided, but the cerebral ones lost nothing of their intensity. The bleeding and leeching were reiterated. The patient remained a few hours in the same state. Blisters were then applied to the legs, and the symptoms were greatly mitigated for four or five days, at the expiration of which the fever and delirium returned, without any other particular symptom. Leeches were again applied to the neck, and blisters to the thighs, which again subdued the fever and cerebral symptoms. The patient passed a fortnight in a tranquil state, but the appetite and strength did not return to their natural range. A diarrhœa now came on, with cough and fever, against which various remedies were unavailing. The strength daily declined, but there was no head-ache, nor intellectual disorder of any kind. It was ascertained by the stethoscope, rather than by the respiration or cough, that organic disease existed in the left side of the chest. The diarrhœa continued, and the patient died at the end of six weeks from the commencement of his complaint.

" *Dissection.* The arachnoid covering both hemispheres was thickened and opake, forming a kind of cap for the brain. The arachnoid of the base of the brain was healthy. There was very little serosity in the ventricles or in any part of the head. The right lung was healthy, but the left was so studded with tubercles that scarce a vestige of its natural organization could be found. There was nothing remarkable in any other part of the body." 519.

We think the above case offers very good evidence that arachnitis had taken place, but that the patient's death was not thereby occasioned. Whether, if the patient had not died of other disease, the thickening and opacity of the arachnoid would have been removed by the powers of Nature, is a question that we cannot answer in our present state of knowledge.

Twelve or fifteen successful cases are detailed by our authors, among which there are several where the cold affusion was employed as an auxiliary to bleeding, and apparently with very good effects. Our limits prevent us from noticing any of these cases, as we are anxious to dedicate a few pages to the following important subject.

VII. *Inflammation of the Spinal Arachnoid.* Our authors neither advocate nor condemn the theories which have, of late years, attributed tetanus, hydrophobia, and certain spasmodic affections to arachnoid inflammation. They wisely and modestly adhere to facts, and ground no opinions upon any other basis.

By a singular and unfortunate concourse of circumstances, it has not occurred to our authors to see a single case of *spinal* arachnitis, unaccompanied by inflammation, more or less, of the arachnoid of the brain. This might seem a formidable embarrassment in the way of establishing a diagnosis between spinal and cerebral arachnitis; but if we find that, in the complications of the two diseases, there were always some peculiar additional symptoms to those where the cerebral arachnoid *only* was inflamed, we may reasonably attribute these additional phenomena to the spinal affection. Several friends, too, of our authors, on whom they could rely with confidence, have met with cases of pure and uncomplicated spinal arachnitis, the symptoms of which perfectly corresponded with those which our authors have traced in the more complicated cases, as appertaining to the disease under consideration.

The most remarkable feature in spinal arachnitis is *contraction of the muscles on the posterior part of the trunk*—a contraction varying from a simple rigidity to the most violent opisthotonos, such as we meet with in tetanus. This muscular contraction must not be confounded with that, which not unfrequently appears in cerebral arachnitis, especially when the inflammation is situated about the base of the brain, and consists in a throwing back of the head, and cervical portion of the spine, while the remainder of the column continues in its natural position. In spinal arachnitis, on the other hand, the whole vertebral column is formed into a kind of inflexible arch, such as our authors never observed in simple inflammation of the cerebral arachnoid.

Next, in order of diagnostic importance to this opisthotonic contraction, is *pain* in some part of the back, varying in seat and degree, but generally most severe in the part corresponding to the site of the inflammation. It also shews remissions, and occasionally complete intermissions. In a very few cases, where cerebral arachnitis was complicated with spinal, the patients did not complain much of pain in the back; but then they were labouring under severe cephalalgia, and our authors very truly remark that violent pain in one organ or part of the body, will often mask affections of other organs or parts; or so absorb the patient's attention as to render him inattentive to any other sensation.

The arching backwards of the spinal column, then, and pain in the back, are the only diagnostic symptoms on which our authors place any reliance. In one patient only they remarked violent pain in the lower extremities. The treatment of this disease must consist in general and local bleeding, and counter-irritants—but it has been very generally fatal in the hands of our continental brethren. We shall now proceed to lay before our readers a selection of such cases as appear to us best calculated to make useful impressions on the practitioner's mind.

*Case* XIV. " Leger, a terrace maker, 28 years of age, of athletic form and strong constitution, had been exposed to great fatigues and bad weather, in demolishing the castle of St. Ouen, near Paris. On the first or second day of December, 1816, he was wounded on the anterior and inner side of the right foot by a nail, which he extracted, and then continued his work. On the 7th December he felt pains in his back. On the 10th he felt an unusual degree of lassitude, while a slight trismus prevented the free exercise of mastication. On the 12th there was some difficulty experienced in bending the spine; yet still LEGER persevered in his daily labours, and even enjoyed some sleep by night. On the 13th, however, LEGER found himself unable to walk, and therefore took to his bed, experiencing the most acute pains in his back. In the night the stiffness of the trunk and the trismus increased—the pains in the back and neck were so excruciating as to force the patient to cry aloud. On the 14th at noon he was conveyed to the HOTEL DIEU, the vertebral column bent into an arch backwards—the jaws incapable of extension to more than half an inch—sense of great constriction in the chest. The original wound in the foot was incised, and some ichorous pus discharged—then a poultice applied. He was bled from the arm—had six grains of opium administered—and was put into the warm bath. A general perspiration ensued, but the pains were exasperated. Laudanum and assafœtida glysters were thrown up—a warm bath, of two hours, produced temporary mitigation of the symptoms. The 15th presented all the phenomena of tetanus, in the form of opisthotonos and trismus, and death closed the scene at two o'clock, P. M.

*Dissection.* The arachnoid covering the upper portions of the cerebral hemispheres presented red patches irregularly circumscribed. At the basis cerebri the arachnoid was sound, and very little serosity was found in the ventricle. The membranes enveloping the spinal marrow were uniformly, and throughout their whole extent and substance, of an extremely vivid red colour. The same appearances were observable in the internal tunics of the cavities of the heart, and of all the large vessels, veins, and arteries. The whole of the blood was fluid in the vessels. All the other organs in the body were sound. The original wound was examined, and the filaments of the plantar nerves traced as far as possible, but without discovering any lesion of structure whatever."

The above case we consider to be one of traumatic tetanus, and in the few dissections which we have made of tetanic patients, we have always found inflammatory traces in the membranes either of the brain or spine. Whether the inflammation in the internal tunics of the heart and vessels were mere coincidences in this case, or had any connexion with the tetanic symptoms, we cannot presume to determine. It may be remarked that, in this case, the cerebral arachnitis was not accompanied by its usual phenomena—a circumstance that may be reasonably accounted for by the intensity of the spinal disease, and the great sufferings of the patient.

*Case* XV. " S. André, 14 years of age, caught a severe cold by having his hair cut too close. Fifteen days after the commencement of the catarrh, he was seized with violent cephalalgia, accompanied with dull pains in the limbs and soles of the feet. His appetite continued good, and these symptoms were dissipated by rest and diluent drinks. After a fit of indigestion, however, the head-ache returned in as much violence as ever, with intense redness of the face, considerable fever, perspirations, quick and precipitous breathing. In this state he continued three days, without medical assistance, and on the 4th day was brought to our authors, exhibiting the following phenomena :—The patient lay on his back, the head forcibly carried backwards and to the left side—the spine arched rigidly, and forming complete opisthotonos—face red, and countenance expressive of great suffering—eyes half open, and the patient not taking notice unless strongly excited—conjunctivæ injected—pupils dilated, especially the right one, but still contractile to the light—great stupor—when roused, with difficulty, from this stupor, he complained of violent occipital cephalalgia, buzzing in the ears, and ocular spectra. He recognized his parents for a few minutes and then fell into a state of reverie and stupor. The lower extremities and left arm were flaccid and even paralytic—the breathing excessively quick and difficult, 41 in the minute—action of the heart strong and regular, but not at all corresponding with the pulse, which was small, vibrating, and 120 in the minute. In the evening there was exacerbation of all the symptoms, with complete loss of sense. *Fifth day*, stupor still more profound—opisthotonos continues—complete paralysis of the left arm, rigidity of the right—pupils dilated and immoveable. In short, all the symptoms became aggravated, and death supervened in the evening.

*Dissection.* Inflammation of that portion of arachnoid situated at the base of the brain, where it was thickened, especially on the tuber annulare, and infiltrated with serum. The arachnoid covering the anterior of the hemispheres presented some patches of inflammation, the ventricles containing two ounces of serum. The vertebral canal being opened completely, and the dura mater slit up, the latter membrane presented a uniform redness throughout its whole extent, the arachnoid covering the spinal marrow being highly inflamed for the space of six or seven inches—the inflamed portion being at a good distance from the head, and covered with a coriaceous exudation. The canal itself contained a quantity of reddish serosity. There were some tubercles in the lungs, and an inflamed portion of peritoneum over the liver." 563.

Our authors consider, and we think justly so, that the opisthotonos was clearly attributable to the spinal arachnitis, especially as there was a considerable space of sound arachnoid intervening between that portion inflamed within the cranium and that within the vertebral canal.

*Case* XVI, " Mary ———, 28 years of age, was violently agitated by an insulting proposal made to her at a time when the menstrual discharge was flowing. The catamenia were suddenly suppressed, and the woman became affected with cold chills for twenty-four hours, succeeded by heat of surface, ardent thirst, and globus hystericus. On the third day, bilious vomiting. On the fourth, the vomitings and hysterical symptoms subsided, and she was received into La Charite, presenting the following phenomena:—*fifth day*, face flushed—eyes brilliant and sparkling—neck tumefied—head thrown backwards—constant pain along the whole line of the vertebral canal exasperated by the slightest motion, (not by pressure,) so as to cause the patient to cry aloud—breathing confined and panting—pulse sharp and quick—skin hot and dry—no stool for four days. Fifteen leeches to the anus—purgative lavement—mustard pediluvia—frictions with liniments to the spine. *Sixth day*, diminution of the symptoms. Blisters to the nape of the neck, and

24 leeches behind the ears. *Seventh day*, some disturbed sleep. The sensibility of the head and back exalted—tetanic rigidity of the neck and spine great—respiration more distressing than ever—face pale and expressive of great suffering. Venesection from the arm, blister to the sacrum, sinapisms, lavements, &c. In three minutes after it was drawn the blood was covered with a thick buff, and strongly cupped. In an hour the patient felt better. The bleeding was therefore reiterated. *Eighth day*, the amelioration continues. Another venesection. *Ninth day*, the blood was buffed and cupped as much as ever; yet the patient is not so well to day. The pain in the head and back is more acute—the features are shrunk, and much altered. Forty-eight leeches were placed as a cordon along the spine on both sides of the vertebræ. These measures produced no relief, and the patient died next day at noon.

*Dissection.* The vertebral canal being opened, and the dura mater incised, a layer of whitish, opake, and membrane-form materials, was found covering the whole spinal marrow, from the occipital hole to the sacrum. On pressing this sheath a fluid was made to flow back into the cranium, On scraping it with a scalpel nothing was separated from it, it was so firm and polished. The arachnoid and pia mater were very much injected, particularly in the fissura magna sylvii. In the right side there was an albuminous concretion, similar to the albuminous layer in the vertebral canal. Similar concretions were found on the external surface of the right hemisphere, under the tentorium cerebelli, and between the cerebellum and basis cranii. The lateral and third ventricles were greatly distended with a lactescent fluid. Organs of chest and abdomen perfectly sound." 568.

The above is certainly a well marked case, and shewed the characteristic symptoms of spinal arachnitis (pain in the back and opisthotonos) in the clearest manner. The woman was confided at La Charite to the care of M. L'Herminier, and our authors consider the mode of treatment as a perfect model (un veritable modèle) for others to follow. Now we are of a different opinion. On the fifth day, when received into the hospital, with all the symptoms of the most terrible excitement in the system, M. L'Herminier contented himself with applying fifteen leeches to the anus, and exhibiting a lavement! Where is the bleeding to syncope recommended by our authors themselves? The patient was not bled till the 7th day, and then the blood shewed the most decisive marks of inflammation, the symptoms being much mitigated by the bleeding. But it is needless to criticise the treatment employed by our continental neighbours in acute diseases. It is perfectly incomprehensible! How our authors too, in the face of their own instructions, could think of recommending M. L'Hermenier's case as a model of active treatment, we are at a loss to conceive.

The cases which press upon our notice in this division of the work, are so very important and interesting, that we entreat the most serious attention of our readers to them, nor do we deem it necessary to make any apology for extending this article by the introduction of such valuable materials—materials which must otherwise be almost entirely lost to the British public.

*Case* XVII. " Cotier, a soldier in the 32d regiment, had been cured of itch at the St. Louis Hospital, but not yet discharged. On the 8th May, 1814, after a debauch in vinous and spirituous liquors, he fell asleep in an open court, where he lay several hours exposed to the sun-beams. In the evening he was carried into his ward, and his companions reported that he passed a very restless night, often starting out of his bed, and uttering piercing cries. Next mŏrning he presented the following phenomena:—General cephalalgia, intolerance of light and noise, red and dry tongue, nausea, difficult deglutition, tenderness on pressure of the epigastrium, respiration laborious, pulse small and quick, marked stiffness in the neck and trunk. A vein was opened in the arm, and another in the foot; but they furnished very little blood. Towards evening, somnolency when the patient was left quiet, but great agitation when touched. Efforts to get out of bed—piercing cries—convulsions of the facial muscles—confusion of intellect, and extreme difficulty in answering questions. A dozen of leeches to the neck. *Third day*, face flushed—eyes fixed—pupils dilated and immoveable—conjunctivæ injected—involuntary lachrymation—contraction and rigidity of the extensor muscles of the neck and spine, forming opisthotonos—inability to speak—diarrhœa—involuntary dircharge of urine. Twelve leeches to the anus, blisters to the legs. *Fourth day*, tetanic symptoms augmented—subsultus tendinum—pupils still dilated and insensible to the most vivid light—erysipelatous edema of the left side of the face—breathing short and embarrassed—pulse intermitting. Died at three, P. M.

*Dissection.* Vessels of the dura mater gorged with black and coagulated blood—arachnoid membrane almost universally inflamed, being red, thickened, injected, and covered with a sero-purulent exudation, in considerable quantity. The medullary substance of the brain was of a reddish hue, and presented innumerable bloody points when sliced. The ventricles contained between six and seven ounces of watery fluid. On laying open the spinal canal, the same marks of this terrible phlegmasia were evident. The spinal arachnoid was inflamed and thickened throughout its whole extent, and a sero-purulent exudation pervaded all parts of the canal." 571.

In the above case, it will be observed, that the pain along the vertebral canal was not complained of, as in most of the cases already detailed. This may be accounted for, however, by the intense state of disease in the brain, and the consequent derangement of intellect. Indeed, it may be very generally observed, that intense cephalalgia, when it exists, absorbs greatly the attention of the patient, who often neglects to mention other pains, unless closely questioned. This ought to be borne in mind.

We shall introduce but one more case, and that principally with the object of admonishing some of our brethren, who would appear to view the vascular system as the grand agent in all maladies, to the almost exclusion of the nerves. We shall perceive, in the following case, several of the characteristics of spinal arachnitis, terminating in death, and yet without presenting a particle of inflammation in head or spine. We shall make some farther reflections after the case is stated.

*Case* XVIII. "A woman, of the name of Roux, 62 years of age, and enjoying habitual good health, experienced a fall, in the middle of April, 1817, which, according to her own account, gave the spinal column and head such a shock as deprived her of sense for a few minutes. From this time till the 29th August following, the occipital region and vertebral column were the seats of constant pain. On the last mentioned day she experienced contractions in the arms, especially the right arm, with great stiffness and rigidity in the neck and trunk—difficult deglutition—pain in the throat, occipital region, and spinal column—tenderness and tension of the abdomen—contraction of the left pupil. The intellectual functions were free; but the tongue was dry and red, with heat of skin, bitter taste in the mouth, and inclination to vomit. The pulse was quick, but void of force. Venesection, warm bath, purgative enema, sulfate of soda in her drink, great restlessness during the night, with pain in the limbs, but no delirium. *Fourth day of these symptoms*, trismus complete—increased rigidity of the left arm—other symptoms the same. Trifling remedies. *Fifth and sixth days*, nearly the same; the tetanic symptoms continued, but the intellects were clear to the last moment. Forty leeches were applied to the spine, but the patient died next day, being the seventh from the commencement of the tetanic symptoms.

*Dissection* shewed the brain and its membranes, the spinal marrow and its coverings, in the most perfect state of integrity, with the exception of a very small quantity of serum in the vertebral canal, and a few varicose vessels near the cauda equina. Every other organ in the body was examined without finding any sign of lesion." 598.

We may observe, on the above case, that the preservation of the intellectual faculties throughout the whole course of the disease was a strong evidence against arachnoid inflammation in the head; but there were most, if not all, the symptoms which accompanied *spinal* arachnitis in numerous other cases. What are we to infer from this? Why, that the ridiculed state called *irritation* of the nervous system will sometimes imitate *inflammation*, and go on to the destruction of life, without leaving a single trace of organic lesion after death. This may teach our younger brethren what a part the nervous system is capable of playing occasionally in the animal economy. At the same time we would be very sorry to advise the practitioner to lean much on the chance of having these nervous irritations to deal with, when the symptoms are equivocal, and there are any reasons to suspect inflammation. In the first place, where the proper phenomena or signs of phlogosis are present, there will be actual inflammation in three cases out of four. In the second place, the mischief arising from treating a neurosis as an inflammation is nothing compared with that which must result from treating a phlegmasia as an irritation or nervous affection. Finally, as we are convinced that nervous irritation is the cousin-german, if not the frequent parent of inflammation, we think it by far the safest practice, in all doubtful cases, to combine the depletive mode of treatment with that which may have a soothing or sedative effect on the nervous system.

We have now brought the analysis of this important work to a close, after expending on it no inconsiderable portion of time and labour. We may again repeat it as our conviction, that the authors of the work before us have taken the only proper mode of investigating a most important class of diseases; and that, although they may not empower us to always distinguish the precise seat of disorder within the head or spine, yet that they will often assist the inexperienced practitioner, and enable him to ascertain, with very considerable probability, not to say accuracy, whether or not he has inflammation of the brain or spine to contend with—a point of knowledge, in the determining of which, the life of his patient and possibly his own reputation may be involved.

We shall take an early opportunity of introducing to the English reader an account of those pathological researches on the diseases affecting the *medullary* substance of the brain, now publishing on the continent, and part of which are already in our possession. In the mean time we entreat the earnest attention of our junior brethren to what we have here presented them.

